# Efficacy and safety outcomes in novel oral anticoagulants versus vitamin-K antagonist on post-TAVI patients: a meta-analysis

**DOI:** 10.1186/s12872-020-01582-2

**Published:** 2020-06-26

**Authors:** Hongbin Liang, Qiyu He, Qiuxia Zhang, Xuewei Liu, Kai Cui, Guojun Chen, Jiancheng Xiu

**Affiliations:** 1 Department of Cardiology, Nanfang Hospital, Southern Medical University, Guangzhou, Guangdong China; 2 Pediatric Cardiac Surgery Center, National Center for Cardiovascular Disease and Fuwai Hospital, Chinese Academy of Medical Sciences, Peking Union Medical College, Beijing, China; 3 Faculty of Medicine, International School, Jinan University, Guangzhou, China

**Keywords:** NOACs, VKA, TAVI, Meta-analysis

## Abstract

**Background:**

Transcatheter aortic valve implantation (TAVI) has been a favored option for the patient who suffered from symptomatic aortic stenosis. However, the efficacy and safety outcomes in novel oral anticoagulants (NOACs) versus Vitamin-K antagonist (VKA) for post-TAVI patients are still controversial. This meta-analysis aims at comparing the clinical outcome and safety of NOACs and VKA in the patients after receiving TAVI.

**Method:**

We searched literature articles in all reachable databases, and observational study as well as randomized controlled trial would be included in order to perform a comprehensive analysis. All-cause mortality, major or life-threatening bleeding, disabling or nondisabling stroke were main pooled outcome measures. Subgroup analysis and meta-regression were adopted to explore heterogeneity. Assessment of bias was performed under the suggestion of Cochrane’s Collaboration Tool.

**Results:**

We collected 3841 non-duplicate citations from PubMed, Embase, Cochrane and ClinicalTrials.gov, and eventually 7 studies were included for this meta-analysis. As a result, VKA showed priority against NOACs in the field of anti-thromboembolism (4435 participants, RR:1.44, 95% CI: 1.05 to 1.99, I^2^ = 0%, *P* = 0.02).

**Conclusion:**

With corroborative analysis of severe complications, VKA is shown to be more protective on post-TAVI patients in disabling or nondisabling stroke scenario but not in mortality or bleeding event.

## Background

Aortic stenosis is one of the valvular diseases and the incidence increases with age. Up to 10% of the population by the eighth decade was affected and the patient would be in danger once the symptoms develop [1]. The annual mortality reaches 25% for symptomatic aortic stenosis, and a majority of patients live no more than 3 years if appropriate treatment cannot be acquired [2]. Transcatheter aortic valve implantation (TAVI) has been reported as a favored option for the patient who suffered from aortic stenosis if the patient do not suitable for surgical replacement [3]. However, bleeding or ischemic events are the predominant issue after TAVI of patients and the optimal antithrombotic therapy post-TAVI remains controversy with the current guideline based on consensus of experts [4]. Plenty of patients undergoing TAVI have comorbidities requiring anticoagulation (OAC) and a combination of antithrombotic agent alongside OAC was administered in most cases [5, 6].

Vitamin-K antagonist (VKA) was a traditional anticoagulant for long-term prevention of thrombosis in the situation of valve replacement [7]. Currently, VKA, as an anticoagulant agent, was recommended for preventing thromboembolic events in post-TAVI patients [8, 9]. However, plenty of limitations of VKA had been reported, such as easily influenced by drugs or foods, and frequent international normalized ratio (INR) monitoring. Novel oral anticoagulants (NOACs) are new medications for preventing or reducing coagulation of blood, which are relatively safer than VKA. Moreover, NOACs were widely adopted in clinical practice as alternatives to VKA in non-valvular atrial fibrillation (AF) with safety profile, while it was reported to be associated with worse outcomes compared with VKA in patients undergoing valve replacement [10–12]. Collectively, either NOACs or VKA is better for post-TAVI patients with indication for anticoagulation remained to be elucidated.

However, there is no certain evidence to perform optimal anticoagulation therapy after cardiac valve replacement, while the current recommendation in cardiovascular practice is predominantly based on expert consensus without adequate evidence [8, 13], especially in TAVI scenario. Consequently, this meta-analysis aims at comparing the clinical outcome of NOACs and VKA in the patients after receiving TAVI and provide the convincing evidence for cardiovascular physician.

## Methods

### Protocol

Preferred Reporting Items for Systematic Reviews and Meta-analyses statement (PRISMA) is as the instruction of this meta-analysis with published retrospective, prospective study, or randomized controlled trial reporting comparison between NOACs and VKA in the patients receiving TAVI. The PRISMA check list is available in Appendix V of supplementary file.

### Eligibility criteria

We included all published study comparing NOACs and VKA among post-TAVI patients, including retrospective, prospective research and randomized controlled trial. Several types of literature were excluded like notes, conference abstract, editorial comment, letter to editor, discussions, systemic review etc. All included patients conformed to the diagnostic criteria of aortic stenosis: maximum aortic jet velocity > 4.0 m/s, mean transvalvular pressure gradient > 40 mmHg, or continuity equation valve area [2], and they all received TAVI.

NOACs belong to a series of drugs and only one of the dabigatran, rivaroxaban, apixaban, edoxaban is treated as intervention in the included study versus VKA (warfarin or its derivates like Phenprocoumon) as the control.

Several specific outcomes measures were extracted for analysis like all-cause mortality, major or life-threatening bleeding, disabling or nondisabling stroke, and combined end points (composite of death from cardiovascular causes, stroke, myocardial infarction, symptomatic valve embolism, deep-vein thrombosis, or systemic embolism). Unpublished data were excluded from our research owing to its unguaranteed quality and validity without peer-review and the overall eligibility criteria were shown in Table 1.

**Table 1:** Eligibility criteria applied in this meta-analysis

	Inclusion Criteria	Exclusion Criteria
Study type	All retrospective or prospective studies.	1. Unfinished studies or unpublished data
		2. Reviews, editorials, letters, notes, discussions, comments, conference abstracts etc.
Participants	Involved patients should conform to the following criteria:	Non-human subjects
	1. Severe aortic stenosis (orifice area < 1 cm^2^), severe co-morbidities that would prohibited surgery.	
	2. Patients in need of OAC.	
	3. Underwent diagnostic evaluation routine laboratory testing, STS score, logistic EuroSCORE, NYHA functional classification, electrocardiography, echocardiography, multislice computed tomography.	
Intervention	Using of at least one of NOACs	N/A
Control	Using of at least one of VKA	N/A
Outcome	1. MACE	Unpublished data
	2. All-caused mortality	
	3. Bleeding	
	4. Disabling or nondisabling stroke	
	5. Combined end-points	

*MACE* Major Adverse Cardiac Events

Combined end-points: Death, stroke, embolism, severe bleeding

### Literature search

We fully searched the PubMed, Embase, Cochrane Central Register of Controlled Trials (CENTRAL), ClinicalTrials.gov until 1st February 2020 under the MeSH terms and searching strategies listed in Appendix I-IV in Supplementary file. Cochrane Highly Sensitive Search Strategy for identifying randomized trials in PubMed and other sources where needed. TAVI, NOACs, VKA were treated as keywords to administer literature searching.

### Study selection

**Fig. 1:**
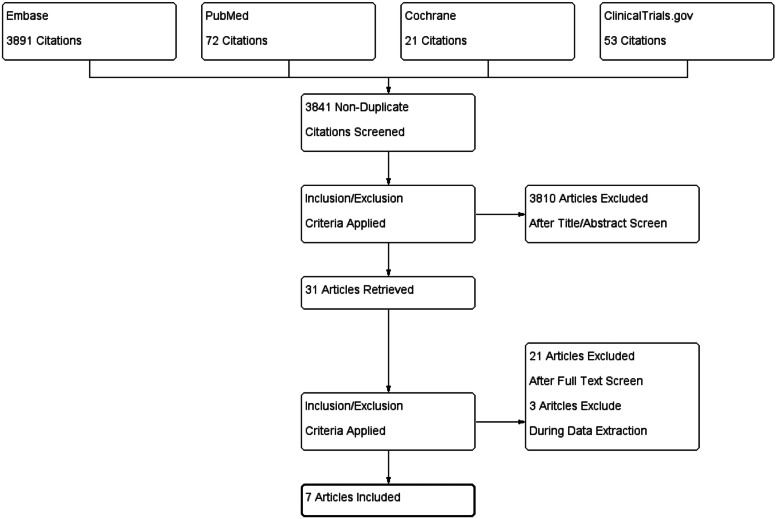
PRISMA flowchart of this meta-analysis

Two authors administered the initial title and abstract screening independently followed by retrieving full text of all eligible studies for further screening. When it comes to disagreement during the process of study selection, a meeting of research group would be appointed for discussion and met the agreement eventually. The overall selection process is demonstrated in a PRISMA flow chart in Fig. 1.

### Data extraction process

Two authors independently started and completed the data extraction process to prevent the phenomenon of test-qualified pooling [14]. Two categories of data were extracted. Firstly, baseline characteristics as gender, age, body mass index (BMI), the co-existing diseases (diabetes mellitus, Chronic obstructive pulmonary disease (COPD), coronary heart disease, history of myocardial infarction, history of cardiac surgery, recent percutaneous intervention, peripheral or cerebral vascular disease, history of stroke or intracranial bleeding, atrial hypertension), CHA2DS2-VASc score, STS Score for mortality, ejection fraction≤50% of the included studies were extracted. Secondly, for outcome measures, all-cause mortality, major or life-threatening bleeding, disabling or nondisabling stroke and combine end-points (composite of death from cardiovascular causes, stroke, myocardial infarction, symptomatic valve embolism, deep-vein thrombosis, or systemic embolism) were pooled for efficacy analysis [15].

### Assessment of heterogeneity

Statistical heterogeneity was analyzed between the included studies via χ2 and I^2^ tests [16], and we set the following criteria of I^2^ > 50% for the existence of heterogeneity, and I^2^ > 70% for the high heterogeneity. Subgroup analysis regarding observational study or RCT was performed in each outcome measure and meta-regression with covariates of age, gender, as well as surgical risks were applicated in the pooled outcomes with existing heterogeneity [17].

### Synthesis of results and analysis

This meta-analysis was performed with RevMan V5.3 and STATA V11.0. For the baseline characteristics, mean and standard deviation (SD) were extracted. If the data was shown in the form of range, then we transformed them to SD via several specific formulas [18–20]. In this meta-analysis, we synthesized dichotomous data by using the number of each event and the risk ratio (RR) and 95% confidence interval (CI) were treated as the principal outcome measures. Besides, the Mantel-Haenszel fixed model was administered when the I^2^<50% while the random model would be performed in the situation of I^2^ > 50% [21]. This meta-analysis was conducted under the consideration of bias and we pooled the data from included studies at low risk of bias. For the follow-up time points, we assessed outcomes at 30 days and 12 months.

### Risk of bias assessment

Two authors independently assessed the risk of bias within the included studies by using the Cochrane Risk of Bias Tool [22] and, the protocols of each study were assessed too. In order to minimize the reporting bias, we completed a comprehensive literature searching process for eligible articles with the adequate MeSH terms and searching quires listed in Appendix I to IV. We assessed the selection bias, performance bias, attrition bias, and reporting bias of included studies and, if multiple follow-up time points existed in one article, each of them would be evaluated respectively. Publication bias was evaluated and quantified by means of egger’s and begg’s test [23, 24]. Besides, funnel plots of each pooled outcome measure were used for visualizing publication bias, and asymmetry would be settled via Harbord’s modified test which is more suitable for dichotomous variables [25].

## Results

### Study selection

We retrieved 72 citations from PubMed, 3891 citations from Embase, 21 citations from Cochrane Library, and 53 citations from ClinicalTrials.gov. After initial title and abstract screening, 31 articles remained with downloaded full text. Two authors assessed the full texts independently and 7 eligible articles were included in this meta-analysis [26–32]. Then we extracted the needed data from these 7 articles with the graphical illustration of the selection process according to the PRISMA statement shown in Fig. 1.

### Study characteristics

This meta-analysis covered a total population of 5089 patients who received TAVI for severe aortic stenosis and were given NOACs and VKA respectively. All of 7 articles, six observational studies and one randomized controlled trial, compared the clinical outcomes between NOACs and VKA. The included researches were clinical controlled study with the average age around 80-year-old, female percentage of about 50%, and the mean BMI ranging from 25.9 to 28.4. Additionally, co-existing diseases or history such as diabetes mellitus (with average of 29.9%), atrial fibrillation (with average of 63.8%) and history of stroke or intracerebral bleeding (with average of 15.5%), which were potential risk factors affecting the end-point of patients, were reported within all included studies. For surgical risks assessment, CHA2DS2-VASc as well as STS risk score were main assessing system, followed by EuroSCORE system. CHA2DS2-VASc score was reported by Seeger et al. [26], Geis et al. [27], Butt et al. [29], Kosmidou et al. [30], as well as GALILEO [32], and STS score system was administered in Seeger et al., Geis et al., Jochheim et al. [28], Butt et al., Kosmidou et al., and GALILEO. However, Kalogeras et al. [31] only adopted Logistic EuroSCORE system in risk assessment. In content of TAVI procedure, several underlying histories of patients apart from the abovementioned diseases should be considered. For instance, coronary artery disease (CAD) and hypertension were reported in three and five studies with the average percentage of 43.2 and 88.9%, respectively. Other demographic and baseline characteristics of included patients were displayed detailedly in Table 2.

**Table 2:** Baseline Characteristics of included populations

Study			Age		Female		BMI		Diabetes Mellitus	
	I	C	NOAC	VKA	NOAC	VKA	NOAC	VKA	NOAC	VKA
Seeger et al., 2017 [26]	Apixaban (*n* = 141)	VKA (*n* = 131)	82.1 (5.3)	80.5 (6.3)	50.4% (71)	48.1% (63)	27.2 (4.2)	27.4 (5.1)	32.6% (46)	32.0% (42)
Geis et al., 2018 [27]	NOAC (*n* = 154)	VKA (*n* = 172)	83.1 (5.3)	83.0 (4.9)	50.6% (78)	54.7% (94)	26.6 (5.3)	27.0 (5.3)	31.0% (47)	33% (57)
Jochheim et al., 2019 [28]	NOAC (*n* = 326)	VKA (*n* = 636)	81.6 (6.7)	81.1 (6.1)	52.1% (170)	52.7% (335)	26.3 (5.2)	26.6 (4.9)	28.8% (94)	34.1% (217)
Butt et al., 2019 [29]	DOAC (*n* = 219)	VKA (*n* = 516)	83.0 (1.8)	82.0 (2.0)	46.1% (101)	46.3% (239)	N/A	N/A	17.8% (39)	24.2% (125)
Kalogeras et al., 2019 [31]	DOAC (*n* = 115)	VKA (*n* = 102)	81.9 (6.3)	82.5 (5.8)	41% (47)	42.2% (43)	27.3 (5.8)	25.9 (5.8)	24.3% (28)	26.8% (28)
Kosmidou et al., 2019 [30]	NOAC (*n* = 155)	VKA (*n* = 778)	82.8 (6.7)		34.4% (321)		28.4 (6.1)		35.3% (330)	
GALILEO, 2020 [32]	NOAC (*n* = 826)	VKA (*n* = 818)	80.4 (7.1)	80.8 (6.0)	48.4% (400)	50.5% (413)	28.1 (5.5)	28.2 (5.7)	28.6% (236)	28.7% (235)
Study	Chronic Renal Failure		COPD		Coronary Heart disease		History of MI		History of Cardiac surgery	
	NOAC	VKA	NOAC	VKA	NOAC	VKA	NOAC	VKA	NOAC	VKA
Seeger et al., 2017 [26]	44.7% (63)	48.9% (64)	N/A	N/A	66.0% (93)	58.8% (77)	17.7% (25)	21.4% (28)	12.8% (18)	12.2% (16)
Geis et al., 2018 [27]	N/A	N/A	N/A	N/A	52.0% (80)	51.0% (88)	N/A	N/A	9.7% (15)	8.0% (13)
Jochheim et al. 2019 [28]	53.3% (174)	44.3% (282)	20.6% (67)	21.9% (139)	N/A	N/A	14.1% (45)	15.4% (94)	8.9% (29)	12.1% (77)
Butt et al., 2019 [29]	5.9% (13)	14.2% (73)	21.0% (46)	16.9% (87)	N/A	N/A	N/A	N/A	N/A	N/A
Kalogeras et al., 2019 [31]	N/A	N/A	N/A	N/A	N/A	N/A	14.8% (17)	10.3% (11)	71.3% (82)	46.1% (47)
Kosmidou et al.,2019 [30]	N/A	N/A	N/A	N/A	N/A	N/A	N/A	N/A	N/A	N/A
GALILEO, 2020 [32]	N/A	N/A	13.3% (110)	10.8% (88)	39.3% (325)	37.3% (305)	N/A	N/A	N/A	N/A
Study	Recent PCI		Haemoglobin pre-TAVI/g/dl		Aortic Valve Area cm2		Peripheral or cerebral vascular disease		History of stroke or intracerebral bleeding	
	NOAC	VKA	NOAC	VKA	NOAC	VKA	NOAC	VKA	NOAC	VKA
Seeger et al., 2017 [26]	N/A	N/A	N/A	N/A	N/A	N/A	82.9% (117)	88.5% (116)	11.3% (16)	14.5% (19)
Geis et al., 2018 [27]	1.0% (2)	1.0% (2)	12.1 (1.8)	11.8 (1.6)	0.7 (0.2)	0.7 (0.2)	N/A	N/A	16.0% (24)	15.0% (25)
Jochheim et al. 2019 [28]	32.5% (106)	29.9% (190)	12.2 (1.8)	12.3 (1.9)	0.7 (0.2)	0.7 (0.2)	4.0% (13)	11.5% (73)	20.6% (67)	21.9% (139)
Butt et al., 2019 [29]	N/A	N/A	N/A	N/A	N/A	N/A	10.1% (22)	8.9% (46)	24.7% (54)	15.1% (78)
Kalogeras et al., 2019 [31]	27% (31)	23.5% (24)	N/A	N/A	0.68 (0.30)	0.67 (0.29)	N/A	N/A	40.2% (41)	31.3% (36)
Kosmidou et al.,2019 [30]	49.1% (458)		N/A		N/A		22% (206)		22.0% (205)	
GALILEO, 2020 [32]	N/A	N/A	N/A	N/A	1.8 (0.6)	1.9 (0.5)	10.0% (83)	10.0% (82)	6.2% (51)	4.3% (35)
Study	Atrial Fibrillation		Hypertension		CHA2DS2-VASc Score		STS Score for mortality		Ejection Fraction≤50%	
	NOAC	VKA	NOAC	VKA	NOAC	VKA	NOAC	VKA	NOAC	VKA
Seeger et al., 2017 [26]	100% (141)	100% (131)	N/A	N/A	5.0 (1.2)	4.9 (1.1)	7.5 (5.2)	7.9 (6.3)	N/A	N/A
Geis et al., 2018 [27]	94.0% (145)	94.0% (161)	95% (147)	92% (158)	4.6 (1.2)	4.8 (1.3)	4.1 (1.9)	4.4 (2.4)	29% (44)	33.0% (57)
Jochheim et al. 2019 [28]	62.0% (202)	75.2% (478)	89.9% (293)	89.5% (569)	≥2:93.6% (*n* = 305)	≥2: 96.1% (*n* = 611)	4.5 (1.1)	4.5 (1.1)	39.9% (130)	39.3% (250)
Butt et al., 2019 [29]	100% (219)	100% (516)	87.2% (191)	88.6% (457)	5.0 (1.4)	4.0 (1.3)	N/A	N/A	N/A	N/A
Kalogeras et al., 2019 [31]	68.7% (79)	59.8% (61)	N/A	N/A	N/A	N/A	N/A	N/A	9.6% (11)	3.9% (4)
Kosmidou et al.,2019 [30]	100% (933)		91.7% (856)		5.6 (1.3)		8.2 (4.2)		N/A	
GALILEO, 2020 [32]	10.4% (87)	11.3% (94)	87.2% (720)	85.2% (697)	4.5 (1.3)	4.6 (1.2)	4.0 (3.2)	4.3 (3.5)	57.4 (11.2)^a^	58.2 (11.2)^1^

Values presented in No. of events (Total) or % (No. of event)

^a^denotes for value (SD)

*I* Intervention

*C* Control

*COPD* Chronic Obstructive Pulmonary Disease

*MI* Myocardial Infarction

*PCI* Percutaneous Cardiac Intervention

Although the included studies focused on comparing the efficacy of NOACs with VKA, antiplatelet therapy was adopted in several patients according to physicians’ discretion. In Seeger et al., antiplatelet therapy would be administered on AF patients 4 weeks after TAVI procedure, while in Geis et al., monotherapy of NOACs or VKA was adopted. Concomitant antiplatelet therapy was employed on patients with previous PCI or known CAD in Jochheim et al. according to discretions of responsible physician. In Kosmidou et al., 57.4% in NOACs group and 58.5% in VKA group were treated with antiplatelet regimen according to current guideline. When it comes to Kalogeras et al., 17.4% patients in NOACs group, and 37.7% patients in VKA group were treated with antiplatelet regimen. In GALILEO, 1.3% patients in control group did not receive antiplatelet therapy due to contraindications. The original data used for calculation were documented in Table 3.

**Table 3:** Original data used for calculation

Study	All-Cause Mortality		Bleeding		Disabling and nondiasbling stroke		Combined end-point^a^		Stroke at early follow-up (30-day)	
	NOAC	VKA	NOAC	VKA	NOAC	VKA	NOAC	VKA	NOAC	VKA
Seeger et al., 2017 [26]	19 (81)	6 (50)	5 (81)	7 (50)	1 (81)	1 (50)	22 (81)	9 (50)	3 (141)	7 (131)
Geis et al., 2018 [27]	12 (154)	11 (172)	3 (154)	3 (172)	5 (154)	2 (172)	17 (154)	14 (172)	N/A	N/A
Jochheim et al., 2019 [28]	47 (326)	70 (636)	69 (326)	146 (636)	10 (326)	13 (636)	63 (326)	87 (636)	5 (326)	6 (636)
Butt et al., 2019 [29]	15 (99)	54 (357)	11 (94)	28 (343)	8 (93)	14 (346)	N/A	N/A	0 (213)	3 (516)
Kalogeras et al., 2019 [31]	13 (115)	16 (102)	6 (115)	9 (102)	N/A	N/A	N/A	N/A	N/A	N/A
Kosmidou et al., 2019 [30]	33 (155)	207 (778)	8 (155)	43 (778)	12 (155)	41 (778)	N/A	N/A	N/A	N/A
GALILEO, 2020 [32]	64 (826)	38 (818)	46 (826)	31 (818)	30 (826)	25 (818)	83 (826)	68 (818)	N/A	N/A

^a^Combined end-points were defined as the composite of death from cardiovascular causes, stroke, myocardial infarction, symptomatic valve embolism, deep-vein thrombosis, or systemic embolism

### Risk of bias assessment

Risk of bias was assessed by two independent authors under the suggestion of Cochrane Collaboration Tool [16] with the detail information and visualization shown in Table S1 and Figure S1. Due to the characteristics of observational studies, selection bias, performance bias and detection bias were high in the Geis et al., Seeger et al., Butt et al. and Kalogeras et al., while the detection bias of Jochhiem et al. was low for the property of prospective study. The exclusion criteria of included 7 studies were stated clearly and the primary outcome measures of interest of these studies were widely accepted for accessing the efficacy of NOACs or VKA on anti-thromboembolic events, making low attrition bias and reporting bias in these articles.

**Fig. 2:**
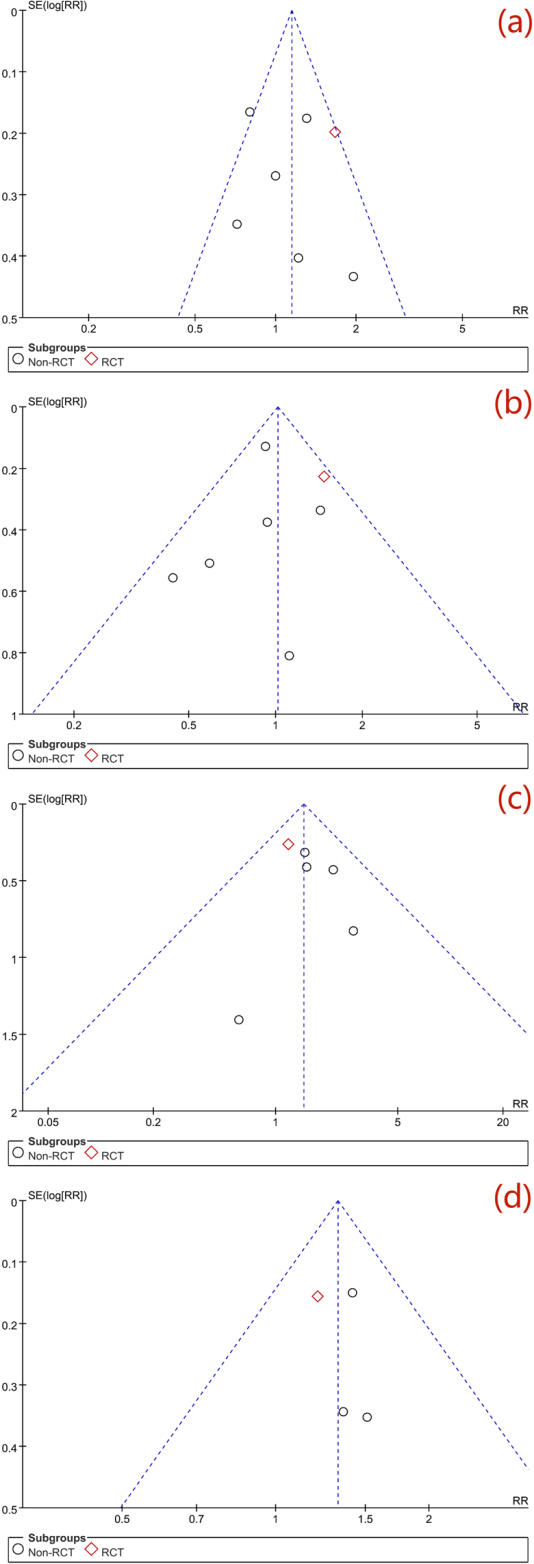
Funnel-plot of assessing publication bias. **a** Funnel-plot of all-cause mortality. **b** Funnel-plot of bleeding. **c** Funnel-plot of disabling or non-disabling stroke. **d** Funnel-plot of combined end-point

In addition, egger’s and begg’s tests were administered to precisely detect the publication bias and, no statistical significance was observed either in egger’s or begg’s test (detailed information was documented in Table S2). Symmetry funnel plots of each outcome measure could be obtained from visual inspection of relevant tests, indicating no publication bias among studies (Fig. 2).

### Study results

#### All-cause mortality

**Fig. 3:**
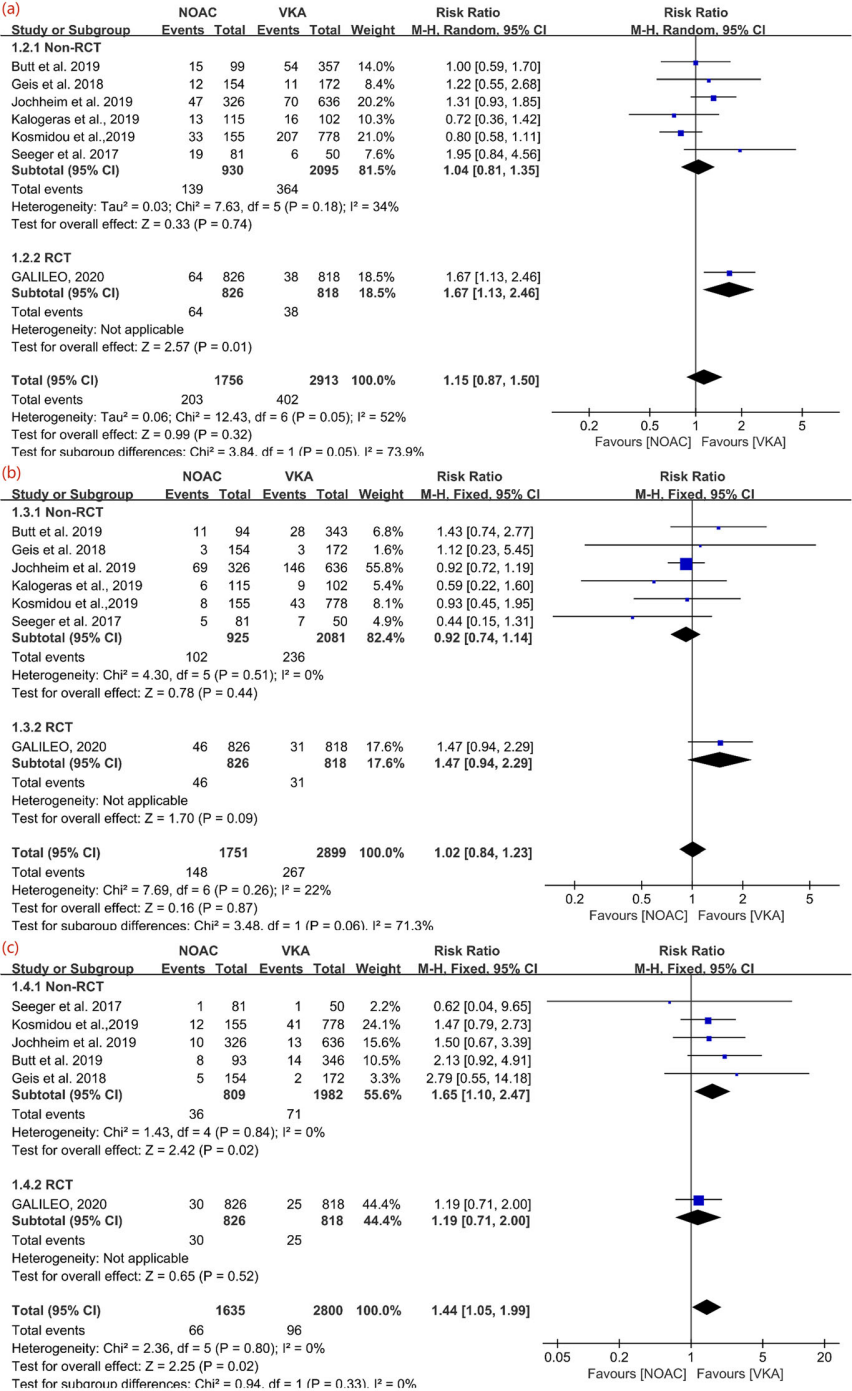
Forrest-plot of each individual outcome. **a** Forrest-plot of all-cause mortality. **b** Forrest-plot of bleeding. **c** Forrest-plot of disabling or non-disabling stroke

For All-cause mortality, Seeger et al., Geis et al., Jochheim et al., Butt et al., Kosmidou et al., Kalogeras et al., and GALILEO were included in the analysis with M-H random model. Within these 7 studies, higher risk of NOACs was only revealed in the subgroup analysis of RCT, GALILEO (1644 participants, RR: 1.67, 95%CI: 1.13 to 2.46), and the other 6 observational studies showed that no statistically significance between NOACs and VKA. To sum up, the pooled estimate indicated that no significant difference in the scenario of all-cause mortality (7 studies, 4669 participants, RR: 1.15, 95% CI:0.87 to 1.50, I^2^ = 52%, *P* = 0.32). The detailed data was displayed in Fig. 3(a).

#### Bleeding

When it comes to the event of major or life-threatening bleeding, Seeger et al., Geis et al., Jochheim et al., Butt et al., Kosmidou et al., Kalogeras et al. and GALILEO were pooled for analysis with Mantel-Haenszel fixed model. Neither the included RCT, GALILEO (1644 participants, RR:1.47, CI: 0.94 to 2.29, *P* = 0.09) nor the remaining observational studies (6 studies, 3006 participants, RR: 0.92, 95% CI: 0.74 to 1.14, I2 = 0, *P* = 0.44) revealed statistically significant difference. The overall results, shown in Fig. 3(b), indicated that no benefit of NOACs compared with VKA in preventing major or life-threatening bleeding (7 studies, 4650 participants, RR:1.02, 95% CI: 0.84 to 1.23, I^2^ = 22%, *P* = 0.87).

#### Stroke

**Fig. 4:**
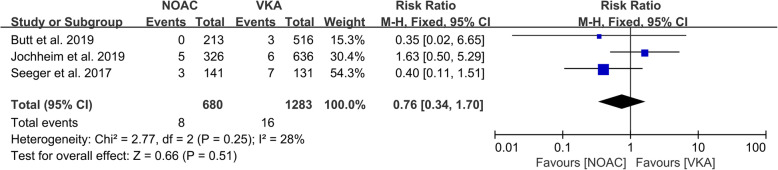
Forrest-plot of stroke occurred in first 30 days after TAVI

For the event of disabling or nondisabling stroke, Seeger et al., Geis et al., Jochheim et al., Butt et al., Kosmidou et al. and GALILEO were included for analysis with Mantel-Haenszel fixed model. No statistical significance could be observed in the subgroup analysis of RCT (1644 participants, RR: 1.19, 95% CI: 0.71 to 2.00, *P* = 0.52), while the subgroup of observational studies revealed protective effect of VKA on post-TAVI patients (5 studies, 2791 participants, RR: 1.65, 95% CI: 1.10 to 2.47, I2 = 0, *P* = 0.02). The overall results showed the better protective effect of VKA compared to NOACs on stroke prevention (6 studies, 4435 participants, RR: 1.44, 95% CI: 1.05 to 1.99, I^2^ = 0%, P = 0.02) with the detailed data referred to Fig. 3(c). Due to the new insights of delayed occurrence of strokes after procedure [33], an extra sub-analysis of follow-up at 30 days focusing on stroke complication was conducted. However, no significantly different protective effect on stroke was observed at 30 days follow-up (3 studies, 1963 participants, RR: 0.76, 95% CI: 0.34 to 1.70, I2 = 28%, *P* = 0.51), with the detailed information showed in Fig. 4. Collectively, as complication, stroke was mainly appended in late follow-up rather than in the first 30 days after procedure.

#### Composite endpoint

**Fig. 5:**
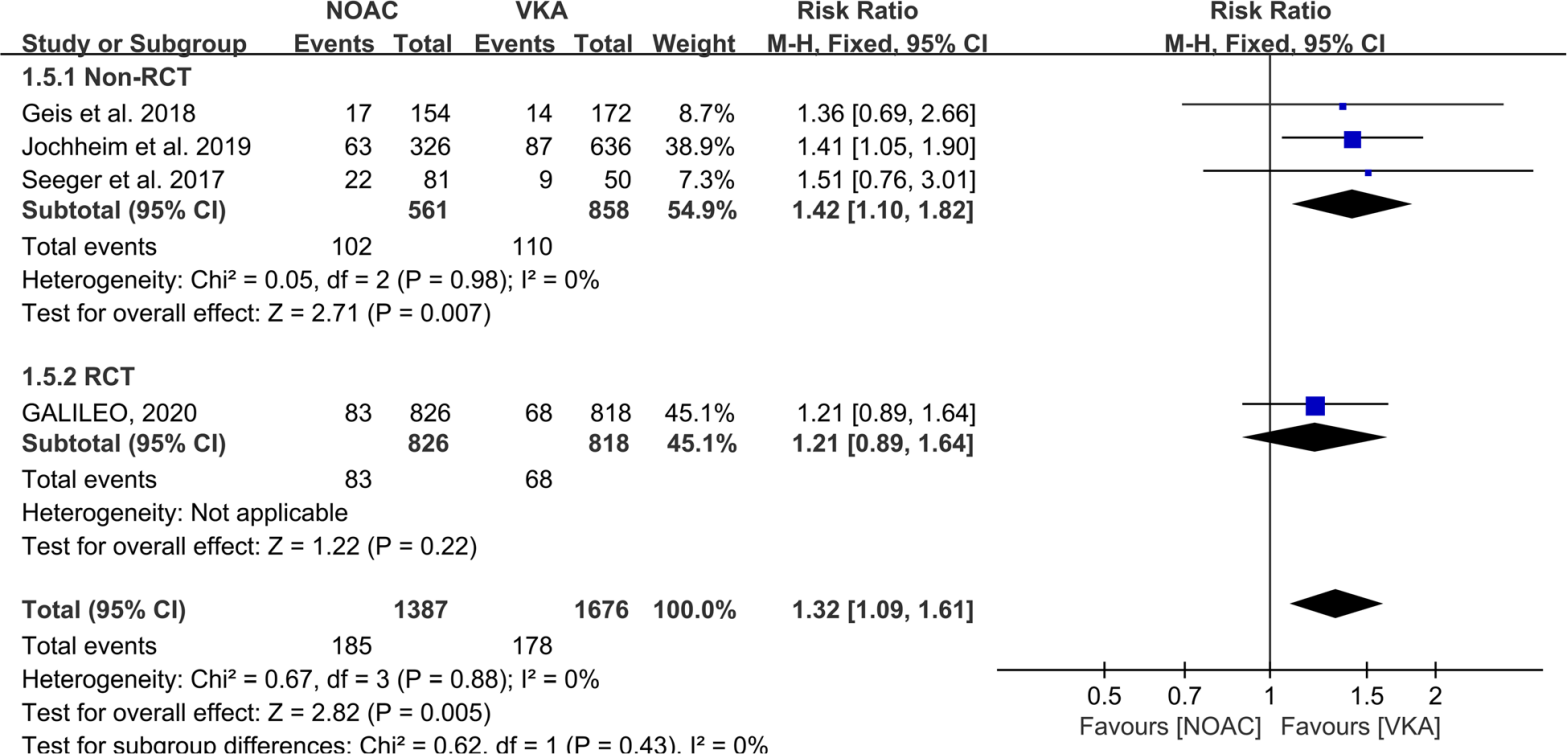
Forrest-plot of combined end-points

In addition, we wanted to determine the anticoagulation strategy in patients with severe complications, followed by analyzing the event of combined end-points (composition of death from cardiovascular causes, stroke, myocardial infarction, symptomatic valve embolism, deep-vein thrombosis, or systemic embolism) with the pooled studies of Seeger et al., Geis et al., Jochheim et al. and GALILEO with Mantel-Haenszel fixed model. Among these analyses, subgroup of RCT revealed the similar effect of NOACs and VKA (1644 participants, RR: 1.21, 95% CI: 0.89 to 1.64, *P* = 0.22), while the protective effect of VKA was prompted by subgroup of observational studies (3 studies, 1419 participants, RR: 1.42, 95% CI: 1.10 to 1.82, I2 = 0%, *P* = 0.007). The overall results recapitulated the protective effect of VKA against NOACs on severe complications prevention (4 studies, 3063 participants, RR: 1.32, 95% CI: 1.09 to 1.61, I2 = 0%, *P* = 0.005). The detailed data was documented in Fig. 5.

### Investigation of heterogeneity

Owing to existence of mild heterogeneity in the analysis of all-cause mortality, bleeding and early stroke at 30 days follow-up, meta-regression was administered to explore the attributable factors. As covariates, age, gender, surgical risks, history of atrial fibrillation and stroke or intracerebral bleeding, which might be potential factors affecting the outcomes of TAVI procedure, were taken into considered. Consequently, none of them was found to be significantly impeding to the results (*P* > 0.05). Detailed information of meta-regression was documented in Table S3.

## Discussion

### Innovation

Nowadays, antithrombotic therapy after TAVI is still controversial and mainly based on expert consensus [4, 8, 13, 34, 35]. Therefore, we performed this most comprehensive meta-analysis, with the first inclusion of RCT, investigating optimal anticoagulation therapy on patient receiving TAVI. For the included 7 studies, Seeger et al. stated that the NOACs was better than VKA in the early safety endpoint and Geis et al., Butt et al. as well as Kalogeras et al. also declared that the NOACs were favored in the situation without additional antiplatelet agents. However, Jochheim et al. and Kosmidou et al. revealed that NOACs and VKA shared the same protective effect on post-TAVI patient. A recently published trial GALILEO [32] included in our study declared the higher risk of NOACs than an antiplatelet-based therapy in scenarios of death or thromboembolism. Collectively, this is the first meta-analysis reporting VKA was shown to be a better regimen compared with NOACs in decreasing risks in post-TAVI patients with inclusion of RCT and adamant evidences.

### Exploration of post-TAVI stroke

In the analysis of all-cause mortality and major or life-threatening bleeding, NOACs and VKA possessed the equivalent protective effect. When it comes to disabling or nondisabling stroke, one of the main complications of post-TAVI patients, VKA showed better protective effect against NOACs. As we known, a majority of patients suffered from severe aortic stenosis are the elder, which is consistent with the baseline characteristics of included studies with the average age around 80. In other words, these populations are frail, elder, or potentially affected by multiple underlying diseases, leading increasing risk of major adverse events after TAVI. In some previous trials investigating anticoagulation therapy with valvular diseases, RE-LY [36] revealed the superiority of NOACs in preventing stroke, bleeding, as well as mortality, and ENGAGE AF-TIMI 48 [37] showed the better protective effect of NOACs compared with VKA in bleeding scenario, which were inconsistent with the recommendation from our results. Thus, a corroborative test specified in analyzing efficacy of NOACs and VKA in severe complications (Fig. 5) was done to convince recommendation of VKA on post-TAVI patients. Not as similar as other valvular diseases ranging from the young to elder, patients needing TAVI are older and risky, which may be the pivotal reason of opposite anticoagulant regimen recommended in previous study or meta-analysis comparing NOACs with VKA [38]. In addition, some concerns come up with leaflet thrombosis (LT) in patients after receiving TAVI, which could contribute to increasing risk of stroke, and it has been elucidated to be reduced by employing anticoagulation therapy [39]. Thus, VKA is recommended for post-TAVI patients in concordance with the results of this study, albeit to the characteristics of lesser periodic blood test and interaction with other drugs of NOACs [40].

### Exploration of heterogeneity

We noticed the mild heterogeneity in analysis of all-cause mortality, bleeding and sub-analysis of stroke at early 30 days follow-up (I2 = 52, 22, 28%, respectively) so that meta-regression for exploring the heterogeneity was adopted. Age, gender, surgical risks, history of atrial fibrillation and stroke or intracerebral bleeding, the essential factors occupied in TAVI, were taken into consideration and no statistical significance was revealed to augment the heterogeneity, indicating that the reported results in this research was not influenced by the abovementioned covariates in meta-regression. As a result, the existing mild heterogeneity might be attributable to not only intrinsic factors of each study but also the other co-existing diseases except cardiovascular system of enrolled patients.

### Limitation and future research

Nevertheless, several limitations should be considered upon this research. For the included studies, several of them were retrospective or prospective studies lacking of randomization which may affect the certainty of results. Besides, the included studies were short of computerized tomography angiography during follow-up resulting in the possibility of missing information on the prevalence of hypo-attenuated leaflet thickening or any other possible differences in two comparable groups. Also, underlying diseases of included patients, interactions between combined anticoagulants, and different arms of NOACs can be the potential factors influencing the assessment of the anti-thrombotic effect so that more trials investigating them are still necessary. Moreover, we noticed that the situation of high-diversity of antithrombotic regimens after receiving TAVI existed, ranging from OAC alone to triple therapy applying OAC in combination with DAPT [27], which hints an additional antiplatelet regimen may improve the prognosis of patients and future study investigating this field is needed. Furthermore, there are two ongoing pivotal randomized controlled trials ATLANTIS comparing apixaban with standard care (NCT02664649) and ENVISAGE-TAVI comparing edoxaban with VKA on post-TAVI patients (NCT02943785), which may reveal more substantial evidences in the field of anticoagulation on post-TAVI patients.

## Conclusion

The risk of VKA on post-TAVI patients in preventing the disabling or nondisabling stroke and combined end-points (severe complications) is lower than NOACs according to our study results, while the optimal anticoagulation management should be made under comprehensive assessment of patient’s status and physician’s discretion in case to case, and further precise randomized controlled trials investigating more scenarios were needed.

## Supplementary information

**Additional file 1: Figure S1**. Visualization of Risk of bias.

**Additional file 2: Table S1.** Risk of bias assessment.

**Additional file 3: Table S2.** Test for publication bias.

**Additional file 4: Table S3.** Data of meta-regression of covariates.

**Additional file 5.**

## Data Availability

All original data used in this study can be found in referred articles.

## Abbreviations

TAVI: Transcatheter aortic valve implantation
NOACs: Novel oral anticoagulants
VKA: Vitamin-K antagonist
INR: International normalized ratio
AF: Atrial fibrillation
PRISMA: Preferred reporting items for systematic reviews and meta-analyses
CENTRAL: Cochrane Central register of controlled trials
COPD: Chronic obstructive pulmonary disease
BMI: Body mass index
SD: Standard deviation
RR: Risk ratio
CI: Confidence interval
CAD: Coronary artery disease
LT: Leaflet thrombosis
