# Supplementary figures and images for: Efficacy and safety outcomes in novel oral anticoagulants versus vitamin-K antagonist on post-TAVI patients: a meta-analysis
Source: BMC Cardiovasc Disord. 2020 Jun 26;20:307. doi: 10.1186/s12872-020-01582-2 (PMC7318737; doi:10.1186/s12872-020-01582-2)

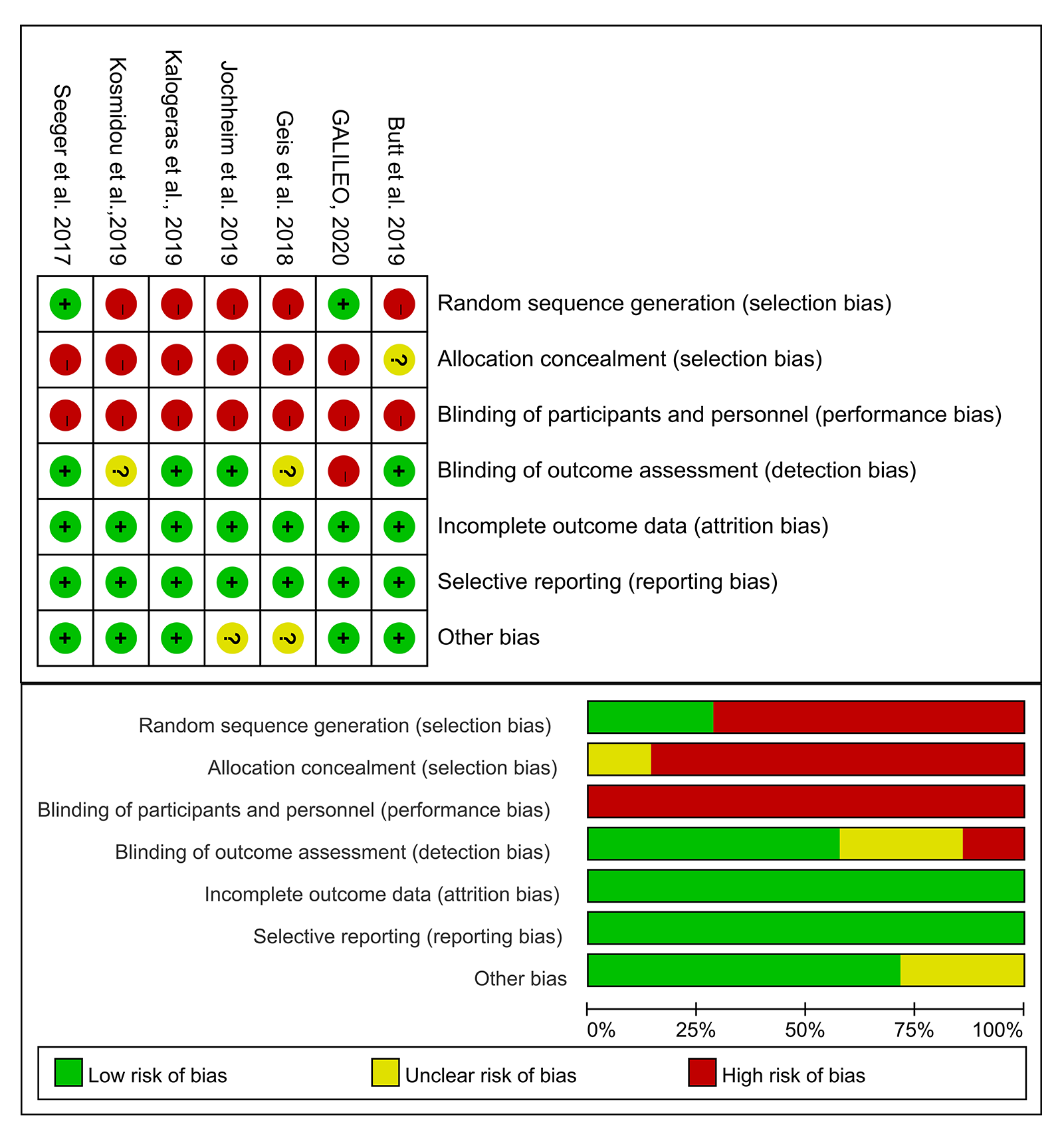

Supplement: Supplementary file 1 — Additional file 1: Figure S1. Visualization of Risk of bias. [file 12872_2020_1582_MOESM1_ESM.png]
