# Supplementary material for: Efficacy and safety outcomes in novel oral anticoagulants versus vitamin-K antagonist on post-TAVI patients: a meta-analysis
Source: BMC Cardiovasc Disord. 2020 Jun 26;20:307. doi: 10.1186/s12872-020-01582-2 (PMC7318737; doi:10.1186/s12872-020-01582-2)
Supplement: Supplementary file 2 — Additional file 2: Table S1. Risk of bias assessment. [file 12872_2020_1582_MOESM2_ESM.docx]

**Table S1. Risk of bias assessment**

| Bias | Judgement | Support |
| --- | --- | --- |
| Article 1 | **Seeger et al., 2017** | |
| Selection | High | Randomization of patients was performed, and this is an open-label study. |
| Performance | High | Patients and clinicians were not blinded. |
| Detection | Low | Assessment was performed without any disturbance. |
| Attrition | Low | The reason of exclusion of patient was clearly stated |
| Reporting | Low | All reported measures of interest are widespread accepted to access the outcomes of patients. |
| Article 2 | **Geis et al., 2018** | |
| Selection | High | This is a retrospective observational study. |
| Performance | High | Patients and clinicians were not blinded. |
| Detection | Unclear | It was not described. |
| Attrition | Low | The reason of exclusion of patient was clearly stated |
| Reporting | Low | All reported measures of interest are widespread accepted to access the outcomes of patients. |
| Article 3 | **Jochheim et al., 2019** | |
| Selection | High | Quote: “This is an investigator initiated multicenter observational registry study conducted in 4 European centers. Only patients in need of oral anticoagulation who underwent TAVR procedure…” |
| Performance | High | Paitents and investigator are aware of group assigned. |
| Detection | Low | This is a prospective study. |
| Attrition | Low | The reason of exclusion of patient was clearly stated |
| Reporting | Low | All reported measures of interest are widespread accepted to access the outcomes of patients. |
| **Article 4** | **Butt et al., 2019** | |
| Selection | High | Quote “All Danish citizens undergoing first-time TAVI between January 1, 2012 and June 30, 2017 were identified…” |
| Performance | High | Patients and clinicians were not blinded. |
| Detection | Low | Assessment was performed without any disturbance. |
| Attrition | Low | The reason of exclusion of patient was clearly stated. |
| Reporting | Low | All reported measures of interest are widespread accepted to access the outcomes of patients. |
| Article 5 | **Kalogeras et al., 2019** | |
| Selection | High | Quote “Consecutive patients, who underwent TAVI…were retrospectively studied.” |
| Performance | High | Patients and clinicians were not blinded. |
| Detection | High | This is a retrospective study. |
| Attrition | Low | The reason of exclusion of patient was clearly stated |
| Reporting | Low | All reported measures of interest are widespread accepted to access the outcomes of patients. |
| Article 6 | **Kosmidou et al.,2019** | |
| Selection | Low | Randomization was administered during the selection process. |
| Performance | High | Patients and clinicians were not blinded. |
| Detection | Unclear | This is a retrospective study and it is not described. |
| Attrition | Low | The reason of exclusion of patient was clearly stated |
| Reporting | Low | All reported measures of interest are widespread accepted to access the outcomes of patients. |
| Article 7 | **GALILEO, 2020** | |
| Selection | Low | Quote “GALILEO was a randomized, controlled trial to evaluate the efficacy and safety of rivaroxaban…anticoagulation. |
| Performance | High | Masking was not available (open label). |
| Detection | High | Masking was not available (open label). |
| Attrition | Low | The reason of exclusion of patient was clearly stated |
| Reporting | Low | All reported measures of interest are widespread accepted to access the outcomes of patients. |
