# Supplementary material for: Efficacy and safety outcomes in novel oral anticoagulants versus vitamin-K antagonist on post-TAVI patients: a meta-analysis
Source: BMC Cardiovasc Disord. 2020 Jun 26;20:307. doi: 10.1186/s12872-020-01582-2 (PMC7318737; doi:10.1186/s12872-020-01582-2)
Supplement: Supplementary file 3 — Additional file 3: Table S2. Test for publication bias. [file 12872_2020_1582_MOESM3_ESM.docx]

**Table S2. Test for publication bias.**

|  | Begg’s Test |  | Egger’s Test |  |  |
| --- | --- | --- | --- | --- | --- |
|  | Z | Pr > \|Z\| | t | P > \|t\| | 95% CI |
| All-Cause Mortality | 0.60 | 0.548 | 0.27 | 0.800 | -211.04 to 260.11 |
| Bleeding | 0.90 | 0.368 | -1.42 | 0.214 | -395.12 to 113.49 |
| Disabling or non-disabling stroke | 0.75 | 0.452 | -1.36 | 0.245 | -570.72 to 195.33 |
| Combined end-point | 0.34 | 0.734 | 0.27 | 0.813 | -260.30 to 295.07 |
| Stroke at 30days after TAVI | 0.00 | 1.000 | -0.80 | 0.571 | -16282.34 to 14357.35 |
