# Supplementary material for: Efficacy and safety outcomes in novel oral anticoagulants versus vitamin-K antagonist on post-TAVI patients: a meta-analysis
Source: BMC Cardiovasc Disord. 2020 Jun 26;20:307. doi: 10.1186/s12872-020-01582-2 (PMC7318737; doi:10.1186/s12872-020-01582-2)
Supplement: Supplementary file 4 — Additional file 4: Table S3. Data of meta-regression of covariates. [file 12872_2020_1582_MOESM4_ESM.docx]

**Table S3. Data of meta-regression of covariates.**

|  | | Coefficient | t | P > \|t\| | 95% Confidence Interval |
| --- | --- | --- | --- | --- | --- |
| Mortality | |  |  |  |  |
|  | Age | -0.1815 | -0.66 | 0.630 | -3.6929 to 3.3300 |
|  | Gender | 1.1193 | 0.59 | 0.661 | -23.0530 to 25.2918 |
|  | Risk scoring | -0.0005 | -0.16 | 0.897 | -0.3865 to 0.3767 |
|  | History of AF | 0.2207 | 0.37 | 0.776 | -7.4102 to 7.8516 |
|  | History of TE | -1.613 | -0.90 | 0.535 | -24.4980 to 21.2723 |
|  | History of DM | 0.0802 | 0.03 | 0.981 | -8.1031 to 8.2634 |
| Bleeding | |  |  |  |  |
|  | Age | 0.4161 | 0.67 | 0.623 | -7.4402 to 8.2724 |
|  | Gender | 1.0828 | 0.20 | 0.877 | -69.2843 to 71.4499 |
|  | Risk scoring | -0.0033 | -0.04 | 0.973 | -0.9875 to 0.9809 |
|  | History of AF | -0.8642 | -0.68 | 0.619 | -16.9779 to 15.2494 |
|  | History of TE | -2.3779 | -0.52 | 0.696 | -60.8056 to 56.0498 |
|  | History of DM | -3.7937 | -1.26 | 0.264 | -11.5378 to 3.9504 |
| Stroke at 30 days | |  |  |  |  |
|  | Age | -0.9779 | -0.54 | 0.686 | -24.0725 to 22.1166 |
|  | Gender | 32.8713 | 1.53 | 0.368 | -239.9864 to 305.7289 |
|  | Risk scoring | -0.2607 | -1.43 | 0.389 | -2.5827 to 2.0613 |
|  | History of AF | -4.7725 | -1.62 | 0.352 | -42.2338 to 32.6887 |
|  | History of TE | 15.9655 | -1.50 | 0.375 | -119.7008 to 151.6317 |
|  | History of DM | 8.6137 | 0.48 | 0.717 | -220.8002 to 238.0276 |

Abbreviations: AF, atrial fibrillation; TE, thromboembolism; DM, diabetes mellitus.
