# Supplementary material for: Efficacy and safety outcomes in novel oral anticoagulants versus vitamin-K antagonist on post-TAVI patients: a meta-analysis
Source: BMC Cardiovasc Disord. 2020 Jun 26;20:307. doi: 10.1186/s12872-020-01582-2 (PMC7318737; doi:10.1186/s12872-020-01582-2)
Supplement: Supplementary file 5 — Additional file 5. [file 12872_2020_1582_MOESM5_ESM.doc]

# Appendix I MeSH terms

# TAVI: Transcatheter Aortic Valve Implantation; Transcatheter Aortic Valve replacement.

# Warfarin: 4-Hydroxy-3-(3-oxo-1-phenylbutyl)-2H-1-benzopyran-2-one; Apo-Warfarin; Aldocumar; Gen-Warfarin; Warfant; Coumadin; Marevan; Warfarin Potassium; Potassium, Warfarin; Warfarin Sodium; Sodium, Warfarin; Coumadine; Tedicumar.

# NOAC: Novel oral anticoagulant; Factor Xa Inhibitor; Non vitamin K anti-coagulant.

# Rivaroxaban: 5-chloro-N-(((5S)-2-oxo-3-(4-(3-oxomorpholin-4-yl)phenyl)-1,3-oxazolidin-5-yl)methyl)thiophene-2-carboxamide; Xarelto; BAY 59-7939; BAY 59 7939; BAY 597939.

# Apixaban: BMS 562247; BMS562247; BMS-562247; Eliquis.

# Edoxaban: N-(5-chloropyridin-2-yl)-N'-((1S,2R,4S)-4-(N,N-dimethylcarbamoyl)-2-(5-methyl-4,5,6,7- tetrahydro(1,3)thiazolo(5,4-c)pyridine-2-carboxamido)cyclohexyl)oxamide; N-(5-chloropyridin-2-yl)-N'-((1S,2R,4S)-4-(N,N-dimethylcarbamoyl)-2-(5-methyl-4,5,6,7-tetrahydrothiazolo(5,4-c)pyridine-2-carboxamido)cyclohexyl)ethanediamide p-toluenesulfonate monohydrate; DU-176b; DU-176; edoxaban tosylate.

# Dabigatran: N-((2-(((4-(aminoiminomethyl)phenyl)amino)methyl)-1-methyl-1H-benzimidazol-5-yl)carbonyl)-N-2-pyridinyl-beta-alanine; BIBR 1048; Pradaxa; Dabigatran Etexilate; Etexilate, Dabigatran; Dabigatran Etexilate Mesylate; Etexilate Mesylate, Dabigatran; Mesylate, Dabigatran Etexilate.

# Appendix II PubMed Search Query

(“Transcatheter aortic valve replacement”[Mesh] OR “Transcatheter aortic valve implantation” OR “aortic valve replacement” OR “transfemoral aortic valve replacement” OR “transfemoral valve implantation”) AND (“NOAC” OR “Novel oral anticoagulant” OR “Factor Xa Inhibitor” OR “Non vitamin k anticoagulant” OR “Oral anticoagulant” OR “Direct oral anticoagulant” OR “Rivaroxaban” OR “5-chloro-N-(((5S)-2-oxo-3-(4-(3-oxomorpholin-4-yl)phenyl)-1,3-oxazolidin-5-yl)methyl)thiophene-2-carboxamide” OR “Xarelto” OR “BAY 59-7939” OR “BAY 59 7939” OR “BAY 597939” OR “Apixaban” OR “BMS 562247” OR “BMS562247” OR “BMS-562247” OR “Eliquis” OR “Edoxaban” OR “N-(5-chloropyridin-2-yl)-N'-((1S,2R,4S)-4-(N,N-dimethylcarbamoyl)-2-(5-methyl-4,5,6,7- tetrahydro(1,3)thiazolo(5,4-c)pyridine-2-carboxamido)cyclohexyl)oxamide” OR “N-(5-chloropyridin-2-yl)-N'-((1S,2R,4S)-4-(N,N-dimethylcarbamoyl)-2-(5-methyl-4,5,6,7-tetrahydrothiazolo(5,4-c)pyridine-2-carboxamido)cyclohexyl)ethanediamide p-toluenesulfonate monohydrate” OR “DU-176b” OR “DU-176” OR “edoxaban tosylate” OR “Dabigatran” OR “N-((2-(((4-(aminoiminomethyl)phenyl)amino)methyl)-1-methyl-1H-benzimidazol-5-yl)carbonyl)-N-2-pyridinyl-beta-alanine” OR “BIBR 1048” OR “Pradaxa” OR “Dabigatran Etexilate” OR “Etexilate, Dabigatran” OR “Dabigatran Etexilate Mesylate” OR “Etexilate Mesylate, Dabigatran” OR “Mesylate, Dabigatran Etexilate”)

# Appendix III EmBase Search Query

**#1:** 'transcatheter aortic valve implantation'/exp OR ‘'transcatheter aortic valve replacement' OR ‘tavi’ OR ‘tavr’ OR ‘transfemoral aortic valve replacement’ OR ‘transfemoral aortic valve implantation’ OR ‘aortic valve replacement’ OR ‘aortic valve implantation’

**#2:** ‘edoxaban’/exp OR ‘du-176b’ OR ‘du-176’ OR ‘edoxaban tosylate’ OR dabigatran’/exp OR 'bibr 1048' OR 'pradaxa' OR 'dabigatran etexilate' OR 'etexilate, dabigatran' OR 'dabigatran etexilate mesylate' OR 'etexilate mesylate, dabigatran' OR 'mesylate, dabigatran etexilate' OR rivaroxaban’/exp OR ‘'xarelto' OR 'bay 59-7939' OR 'bay 59 7939' OR 'bay 597939' OR ‘apixaban’/exp OR 'bms 562247' OR 'bms562247' OR 'bms-562247' OR 'eliquis' OR 'noac' OR 'novel oral anticoagulant'/exp OR 'novel oral anticoagulant' OR 'oral anticoagulant'/exp OR 'oral anticoagulant' OR 'factor xa inhibitor'/exp OR 'factor xa inhibitor' OR 'direct oral anticoagulant'/exp OR 'direct oral anticoagulant' OR 'non vitamin k antagonist'/exp OR 'non vitamin k antagonist'

**#3:** #1 AND #2

# Appendix IV Cochrane Search Query

#1 MeSH descriptor: [Transcatheter Aortic Valve Replacement] explode all trees

#2 (TAVI):ti,ab,kw (Word variations have been searched)

#3 (TAVR):ti,ab,kw (Word variations have been searched)

#4 (aortic valve replacement):ti,ab,kw (Word variations have been searched)

#5 (aortic valve implantation):ti,ab,kw (Word variations have been searched)

#6 (transfemoral aortic valve replacement):ti,ab,kw (Word variations have been searched)

#7 (transfemoral aortic valve implantation):ti,ab,kw (Word variations have been searched)

#8 #1 OR #2 OR #3 OR #4 OR #5 OR #6 OR #7

#9 MeSH descriptor: [Dabigatran] explode all trees

#10 MeSH descriptor: [Rivaroxaban] explode all trees

#11 (Xarelto):ti,ab,kw (Word variations have been searched)

#12 (BAY 59 7939):ti,ab,kw (Word variations have been searched)

#13 (BAY 597939):ti,ab,kw (Word variations have been searched)

#14 (apixaban):ti,ab,kw (Word variations have been searched)

#15 (BMS 562247):ti,ab,kw (Word variations have been searched)

#16 (Eliquis):ti,ab,kw (Word variations have been searched)

#17 (Edoxaban):ti,ab,kw (Word variations have been searched)

#18 (DU-176b):ti,ab,kw (Word variations have been searched)

#19 (DU-176):ti,ab,kw (Word variations have been searched)

#20 (edoxaban tosylate):ti,ab,kw (Word variations have been searched)

#21 (BIBR 1048):ti,ab,kw (Word variations have been searched)

#22 (BIBR 1048):ti,ab,kw (Word variations have been searched)

#23 (BIBR 1048):ti,ab,kw (Word variations have been searched)

#24 (Pradaxa):ti,ab,kw (Word variations have been searched)

#25 (Dabigatran Etexilate):ti,ab,kw (Word variations have been searched)

#26 (Dabigatran Etexilate Mesylate):ti,ab,kw (Word variations have been searched)

#27 #9 OR #10 OR #11 OR #12 OR #13 OR #14 OR #15 OR #16 OR #17 OR #18 OR #19 OR #20 OR #21 OR #22 OR #23 OR #24 OR #25 OR #26

#28 #8 AND #27

**Appendix V** PRISMA Check List

| **Section/topic** | **#** | **Checklist item** | **Reported on page #** |
| --- | --- | --- | --- |
| **TITLE** | | |  |
| Title | 1 | Identify the report as a systematic review, meta-analysis, or both. | 1 |
| **ABSTRACT** | | |  |
| Structured summary | 2 | Provide a structured summary including, as applicable: background; objectives; data sources; study eligibility criteria, participants, and interventions; study appraisal and synthesis methods; results; limitations; conclusions and implications of key findings; systematic review registration number. | 2 |
| **INTRODUCTION** | | |  |
| Rationale | 3 | Describe the rationale for the review in the context of what is already known. | 3 |
| Objectives | 4 | Provide an explicit statement of questions being addressed with reference to participants, interventions, comparisons, outcomes, and study design (PICOS). | 3 |
| **METHODS** | | |  |
| Protocol and registration | 5 | Indicate if a review protocol exists, if and where it can be accessed (e.g., Web address), and, if available, provide registration information including registration number. | 3 |
| Eligibility criteria | 6 | Specify study characteristics (e.g., PICOS, length of follow-up) and report characteristics (e.g., years considered, language, publication status) used as criteria for eligibility, giving rationale. | 3-4 |
| Information sources | 7 | Describe all information sources (e.g., databases with dates of coverage, contact with study authors to identify additional studies) in the search and date last searched. | 4 |
| Search | 8 | Present full electronic search strategy for at least one database, including any limits used, such that it could be repeated. | 4 |
| Study selection | 9 | State the process for selecting studies (i.e., screening, eligibility, included in systematic review, and, if applicable, included in the meta-analysis). | 4 |
| Data collection process | 10 | Describe method of data extraction from reports (e.g., piloted forms, independently, in duplicate) and any processes for obtaining and confirming data from investigators. | 4-5 |
| Data items | 11 | List and define all variables for which data were sought (e.g., PICOS, funding sources) and any assumptions and simplifications made. | 5 |
| Risk of bias in individual studies | 12 | Describe methods used for assessing risk of bias of individual studies (including specification of whether this was done at the study or outcome level), and how this information is to be used in any data synthesis. | 5 |
| Summary measures | 13 | State the principal summary measures (e.g., risk ratio, difference in means). | 5 |
| Synthesis of results | 14 | Describe the methods of handling data and combining results of studies, if done, including measures of consistency (e.g., I^2^) for each meta-analysis. | 5 |

| **Section/topic** | **#** | **Checklist item** | **Reported on page #** |
| --- | --- | --- | --- |
| Risk of bias across studies | 15 | Specify any assessment of risk of bias that may affect the cumulative evidence (e.g., publication bias, selective reporting within studies). | 5 |
| Additional analyses | 16 | Describe methods of additional analyses (e.g., sensitivity or subgroup analyses, meta-regression), if done, indicating which were pre-specified. | 5 |
| **RESULTS** | | |  |
| Study selection | 17 | Give numbers of studies screened, assessed for eligibility, and included in the review, with reasons for exclusions at each stage, ideally with a flow diagram. | 5-6 |
| Study characteristics | 18 | For each study, present characteristics for which data were extracted (e.g., study size, PICOS, follow-up period) and provide the citations. | 6-7 |
| Risk of bias within studies | 19 | Present data on risk of bias of each study and, if available, any outcome level assessment (see item 12). | 6-7 |
| Results of individual studies | 20 | For all outcomes considered (benefits or harms), present, for each study: (a) simple summary data for each intervention group (b) effect estimates and confidence intervals, ideally with a forest plot. | 7-8 |
| Synthesis of results | 21 | Present results of each meta-analysis done, including confidence intervals and measures of consistency. | 7-8 |
| Risk of bias across studies | 22 | Present results of any assessment of risk of bias across studies (see Item 15). | 6-7 |
| Additional analysis | 23 | Give results of additional analyses, if done (e.g., sensitivity or subgroup analyses, meta-regression [see Item 16]). | 8 |
| **DISCUSSION** | | |  |
| Summary of evidence | 24 | Summarize the main findings including the strength of evidence for each main outcome; consider their relevance to key groups (e.g., healthcare providers, users, and policy makers). | 8-9 |
| Limitations | 25 | Discuss limitations at study and outcome level (e.g., risk of bias), and at review-level (e.g., incomplete retrieval of identified research, reporting bias). | 9 |
| Conclusions | 26 | Provide a general interpretation of the results in the context of other evidence, and implications for future research. | 9-10 |
| **FUNDING** | | |  |
| Funding | 27 | Describe sources of funding for the systematic review and other support (e.g., supply of data); role of funders for the systematic review. | 10 |

*From:*  Moher D, Liberati A, Tetzlaff J, Altman DG, The PRISMA Group (2009). Preferred Reporting Items for Systematic Reviews and Meta-Analyses: The PRISMA Statement. PLoS Med 6(7): e1000097. doi:10.1371/journal.pmed1000097

For more information, visit: **www.prisma-statement.org**.
